# Elevated perfusate [Na^+^] increases contractile dysfunction during ischemia and reperfusion

**DOI:** 10.1038/s41598-020-74069-x

**Published:** 2020-10-14

**Authors:** D. Ryan King, Rachel L. Padget, Justin Perry, Gregory Hoeker, James W. Smyth, David A. Brown, Steven Poelzing

**Affiliations:** 1 Translational Biology, Medicine, and Health Graduate Program, Virginia Polytechnic Institute and State University, Blacksburg, VA USA; 2 Department of Human Nutrition, Virginia Polytechnic Institute and State University, Foods, and Exercise, Blacksburg, VA USA; 3 Center for Heart and Reparative Medicine Research, Fralin Biomedical Research Institute at Virginia Tech Carilion, Roanoke, VA 24016 USA; 4 Virginia Tech Carilion School of Medicine, Roanoke, VA USA; 5 Department of Biomedical Engineering and Mechanics, Virginia Polytechnic Institute and State University, Blacksburg, VA USA; 6 Department of Biological Sciences, Virginia Polytechnic Institute and State University, Blacksburg, VA USA

**Keywords:** Physiology, Cardiology, Cardiovascular biology

## Abstract

Recent studies revealed that relatively small changes in perfusate sodium ([Na^+^]_o_) composition significantly affect cardiac electrical conduction and stability in contraction arrested ex vivo Langendorff heart preparations before and during simulated ischemia. Additionally, [Na^+^]_o_ modulates cardiomyocyte contractility via a sodium-calcium exchanger (NCX) mediated pathway. It remains unknown, however, whether modest changes to [Na^+^]_o_ that promote electrophysiologic stability similarly improve mechanical function during baseline and ischemia–reperfusion conditions. The purpose of this study was to quantify cardiac mechanical function during ischemia–reperfusion with perfusates containing 145 or 155 mM Na^+^ in Langendorff perfused isolated rat heart preparations. Relative to 145 mM Na^+^, perfusion with 155 mM [Na^+^]_o_ decreased the amplitude of left-ventricular developed pressure (LVDP) at baseline and accelerated the onset of ischemic contracture. Inhibiting NCX with SEA0400 abolished LVDP depression caused by increasing [Na^+^]_o_ at baseline and reduced the time to peak ischemic contracture. Ischemia–reperfusion decreased LVDP in all hearts with return of intrinsic activity, and reperfusion with 155 mM [Na^+^]_o_ further depressed mechanical function. In summary, elevating [Na^+^]_o_ by as little as 10 mM can significantly modulate mechanical function under baseline conditions, as well as during ischemia and reperfusion. Importantly, clinical use of Normal Saline, which contains 155 mM [Na^+^]_o_, with cardiac ischemia may require further investigation.

## Introduction

In recent years, our laboratory published several manuscripts demonstrating that relatively small changes in perfusate ionic composition, particularly sodium, have profound effects on cardiac electrical conduction and stability, especially when these changes occur in conjunction with a cardiac insult such as ischemia or reduced gap junctional coupling^1–3^. Due to the nature of the optical mapping modality used to study electrophysiology in our previous studies, it remains unknown whether these same modest changes in perfusate ionic composition impact mechanical function.

The concept of altered extracellular or intracellular sodium concentrations ([Na^+^]_o_ or [Na^+^]_i_, respectively) directly influencing cardiac inotropy was first published in 1948^4^. In the subsequent half century, numerous studies focused on exploring sarcolemma sodium-calcium exchange and the sodium-calcium exchanger protein (NCX) itself^5,6^. NCX is a pore forming transmembrane channel that exchanges Na^+^ and Ca^2+^ in a 3-to-1 ratio across the sarcolemma, capable of functioning in both forward (Ca^2+^-efflux) and reverse (Ca^2+^-influx) modes^7^. Previous research demonstrated that changes in either [Na^+^]_o_ or [Na^+^]_i_ affect the NCX current^6^. Specifically, isolated myocyte studies demonstrated that reducing [Na^+^]_o_ increased Ca^2+^ influx through NCX^8,9^.

Importantly, studies have also demonstrated that ventricular arrhythmias and myofibrillar hypercontracture associated with ischemia–reperfusion injury are, at least in part, due to intracellular Ca^2+^ overload and increased calcium-calmodulin dependent protein kinase II (CaMKII) activity^10,11^. Therefore, it stands to reason that increasing [Na^+^]_o_ should decrease Ca^2+^ influx through NCX, which leads to the specific hypothesis that elevating [Na^+^]_o_ reduces ischemia–reperfusion injury in ex vivo Langendorff-perfused hearts. We herein present an analysis of the mechanical consequences of elevating [Na^+^]_o_ from 145 to 155 mM to compliment previous studies in our laboratory evaluating the effects of hypernatremia on cardiac conduction during ischemia^2,3^. During normal baseline periods, elevating [Na^+^]_o_ depressed left-ventricular developed pressure (LVDP) as hypothesized, but did not influence intrinsic heart rate. During ischemia, hearts exposed to elevated [Na^+^]_o_ demonstrated an earlier onset of ischemic contracture (i.e. a more rapid onset of ischemic mechanical dysfunction). Elevated [Na^+^]_o_ during reperfusion also reduced inotropy below baseline values. In short, [Na^+^]_o_ management may differentially affect electrical and mechanical function of the heart.

## Results

### Baseline: [Na^+^] and left ventricular developed pressure

**Figure 1:**
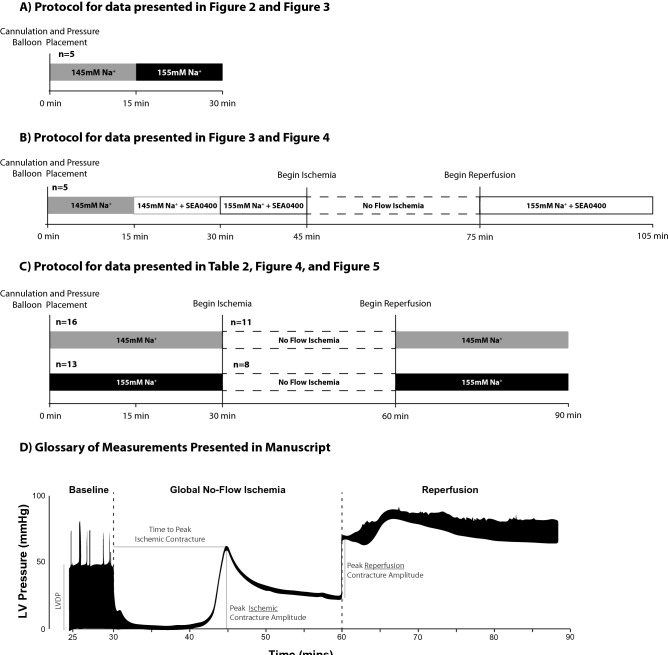
Experimental protocol design. (**A**) Sequential perfusion of 145 mM Na^+^ and 155 mM Na^+^ perfusates (n = 5), data presented in Figs. 2 and 3B. (**B**) SEA0400 perfusion (n = 5), data presented in Figs. 3 and 4. (**C**) Independent perfusion of 145 mM Na^+^ and 155 mM Na^+^ perfusates (n = 16 and n = 13, respectively, for baseline measurements, n = 11 and n = 8 hearts from each group were subjected to the ischemia reperfusion protocol). (**D**) Definitions of experimental measurements. Representative pressure trace from one heart perfused with the 145 Na^+^ perfusate defining left-ventricular developed pressure (LVDP; LVDP = Cyclic systolic pressure maximum – cyclic diastolic pressure minimum), time to peak ischemic contracture, amplitudes of ischemic and reperfusion contracture at return of cardiac perfusion.

Previous studies demonstrated that eliminating or pathologically reducing perfusate sodium concentration ([Na^+^]_o_) to 70–75 mM in ex vivo preparations is positively inotropic^8,9^. Similarly, elevating [Na^+^]_o_ to 162 mM decreases LVDP^12^. However, to our knowledge, no groups have demonstrated the functional effects of varying [Na^+^]_o_ within a clinically relevant range (i.e. 145–155 mM) using a Langendorff-perfused heart model. In this study, the effects of elevated [Na^+^]_o_ on cardiac mechanical function were assessed at baseline and during global ischemia–reperfusion in Langendorff-perfused rat hearts. Diagrams of the experimental protocols (Fig. 1A–C) and a representative trace of left-ventricular pressure (Fig. 1D) are provided in Fig. 1 for the purpose of visualizing pressure prior to, during, and following global no-flow ischemia.

**Figure 2:**
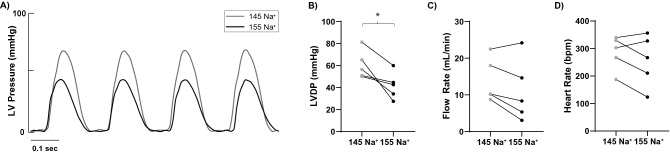
Increasing perfusate Na^+^ depresses LVDP, independent of changes in flow rate or heart rate. (**A**) Representative paired left ventricular (LV) pressure traces from a single heart during 145 mM [Na^+^]_o_ (145 Na^+^) and 155 mM [Na^+^]_o_ (155 Na^+^) perfusion. (**B**) LVDP was significantly reduced in paired comparisons during perfusion with 155 relative to 145 mM [Na^+^]_o_. (**C**) Flow rate was not significantly reduced when hearts were perfused with 155 relative to 145 mM [Na^+^]_o_. (**D**) Heart rate did not significantly change when varying [Na^+^]_o_ during baseline conditions. n = 5, *Indicates p < 0.05 as determined by paired Student’s t-test.

Representative left-ventricular pressure traces obtained during the pre-ischemia (baseline) protocol are provided for 15 min of perfusion with 145 mM Na^+^ followed by an additional 15 min of perfusion with 155 mM Na^+^ (Fig. 2A). Parameters are summarized for the last 5 min of baseline perfusion and reveal that elevating [Na^+^]_o_ significantly decreased mean LVDP by 19.0 ± 12.8 mmHg (Fig. 2B), without altering coronary flow rate (Fig. 2C) or heart rate (Fig. 2D).

To confirm these results were a consequence of increased [Na^+^]_o_ and not a function of perfusion time, baseline measurements were repeated in hearts perfused independently with either the 145 or 155 mM Na^+^ perfusate from the beginning of cannulation. In unpaired comparisons, LVDP was significantly higher in hearts perfused with 145 mM Na^+^ (50.6 ± 16.8 mmHg) relative to 155 mM Na^+^ (28.5 ± 10.6 mmHg), with no significant differences in baseline end-diastolic pressure (EDP), coronary flow rate, or heart rate (Table 2). Together, the data further demonstrate that elevating [Na^+^]_o_ within a near physiologic range, from 145 to 155 mM, decreases LVDP.

As baseline LVDP values may have variability based on the fill state of the balloon, the uniformity of contact with the ventricular wall, and other factors associated with measuring pressure in a non-working isolated heart-model, we conducted all baseline pressure analyses on raw data with paired comparisons where appropriate.

### Baseline: NCX and LVDP

It is well-established that elevating [Na^+^]_o_ increases NCX forward-mode activity (Ca^2+^ efflux) and subsequently decreases LVDP^8,9,13,14^. It is once again important to note that these previous results are based on pathological concentrations of Na^+^ and it therefore remains unknown whether a modest alteration of [Na^+^]_o_ could measurably alter LVDP via an NCX mediated pathway.

**Figure 3:**
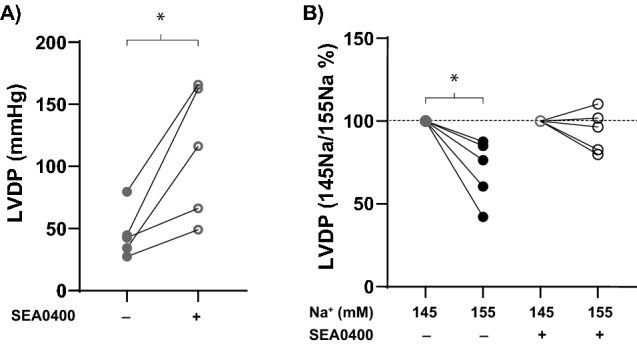
NCX inhibition (with 1 µM SEA0400) mitigates the negative inotropic effect of increasing [Na^+^]_o_. (**A**) NCX inhibition with SEA0400 significantly increases LVDP at baseline with 145 mM Na^+^. (**B**) SEA0400 treatment prior to elevating [Na^+^]_o_ mitigates the negative inotropic effect of elevated [Na^+^]_o_. n = 5, *Indicates p < 0.05 as determined by paired Student’s t-test.

To confirm enhanced NCX forward-mode activity during elevated [Na^+^]_o_ was a mechanism of Na^+^-dependent reduction of LVDP, hearts were treated with the NCX inhibitor SEA0400 (1 µM) prior to elevating [Na^+^]_o_ from 145 to 155 mM^15,16^. As anticipated, in hearts perfused with 145 mM Na^+^ at baseline, NCX inhibition was positively inotropic (Fig. 3A). Furthermore, inhibiting NCX prior to elevating [Na^+^]_o_ mitigated the reduction of LVDP observed without NCX inhibition (Fig. 3B), as evidenced by the steeper decline in LVDP between 145 and 155 mM Na^+^ in the absence versus presence of SEA0400. These results confirm a role for NCX in Na^+^-dependent modulation of LVDP, consistent with previous studies^8,9^.

### Ischemia: [Na^+^] and ischemic contracture

A known phenomenon in rodent hearts is a marked rise in diastolic pressure during global ischemia, termed ischemic contracture^17^. Previous studies demonstrated a marked increase in [Na^+^]_i_ accompanies ischemia, and suggest elevating [Na^+^]_i_ concentration reduces mitochondrial response to stress in cardiomyocytes, resulting in decreased oxidative phosphorylation^13,18,19^. Furthermore, studies have demonstrated the rise in diastolic tone during ischemia is a consequence of improper cross-bridge relaxation due to ATP depletion within the cell^17^. As such, we hypothesized elevating [Na^+^]_o_ before the ischemic episode would increase [Na^+^]_i_ accumulation, further disrupt oxidative phosphorylation, and reduce the time to peak ischemic contracture.

**Figure 4:**
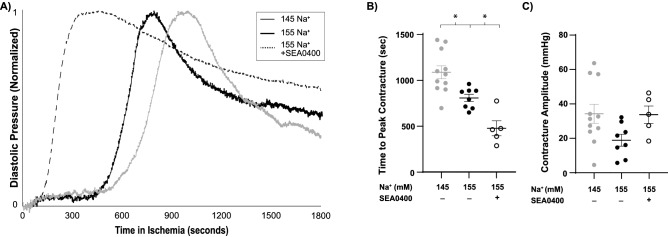
Elevating [Na^+^]_o_ accelerates the onset of ischemic contracture. Pre-ischemic treatment with SEA0400 further accelerates the onset of ischemic contracture. (**A**) Representative traces of left-ventricular pressure during 30-min no-flow global ischemia for the 145 Na^+^, 155 Na^+^, and 155 Na^+^  + SEA0400 groups (amplitude full scale normalized for visualization purposes; EDP’—Eq. (1)). (**B**) Time to peak ischemic contracture for all groups. (**C**) Diastolic amplitude at time of peak ischemic contracture. (n = 11, 8, and 5 for 145 Na^+^, 155 Na^+^, and 155 Na^+^  + SEA0400, perfusates respectively, * indicates p < 0.05 as determined by a one-way ANOVA with Dunnett’s correction for multiple comparisons to the 155 Na^+^ group).

In our experiments, the time course of ischemic contracture was dependent on the pre-ischemic perfusate composition (Fig. 4). More specifically, increasing [Na^+^]_o_ shortened the time to onset of ischemic contracture (Fig. 4A,B). The amplitude of ischemic contracture in Fig. 4A was normalized independently for each trace using Eq. (1) below, where EDP’ indicates the normalized EDP. Representative normalized EDP traces for each perfusate composition were plotted together for visualization purposes (Fig. 4A).

1 $$ EDP^{\prime} = \frac{{EDP - EDP_{\min } }}{{EDP_{\max } - EDP_{\min } }} $$

Inhibiting NCX prior to global ischemia further accelerated the time to onset of ischemic contracture (Fig. 4A,B). The peak amplitude of ischemic contracture was not significantly different in hearts perfused with the 155 mM Na^+^ perfusate prior to ischemia (relative to 145 mM Na^+^, p = 0.06), or with 155 mM Na^+^ and SEA0400 (p = 0.99; Fig. 4C).

### Reperfusion: [Na^+^] and cardiac functional recovery

**Figure 5:**
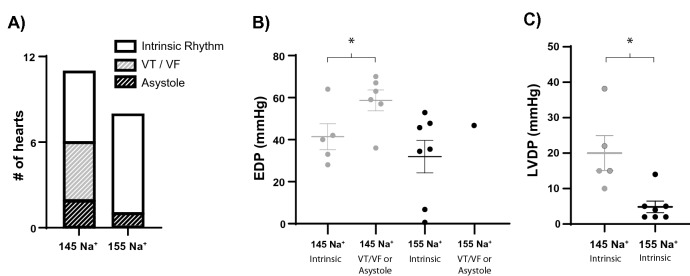
Elevated [Na^+^]_o_ increases contractile dysfunction during reperfusion in hearts with a return of intrinsic rhythm. (**A**) Stacked bar graphs summarizing the return of electrical rhythm during reperfusion. (**B**) Peak EDP immediately upon reperfusion (reperfusion contracture). In the 145 mM Na^+^ group, hearts that eventually returned to an intrinsic rhythm during reperfusion had a significantly lower EDP than those developing arrhythmias or asystole; there was no difference in EDP between the 145 mM Na^+^ and 155 mM Na^+^ groups when only comparing EDP from hearts with returned intrinsic rhythm. (**C**) LVDP at 30-min of reperfusion is significantly higher in the 145 Na^+^ group relative to the 155 Na^+^ group, consistent with baseline LVDP values. Data from arrhythmic and asystolic hearts excluded for (**C**). *Indicates p < 0.05 as determined by unpaired student’s t-test.

Following 30-min of global no-flow ischemia, hearts were reperfused with either the 145 or 155 mM Na^+^ perfusate. Intrinsic rhythm returned in five of eleven hearts perfused with the 145 mM Na^+^ perfusate, and seven of eight hearts perfused with the 155 mM Na^+^ perfusate (Fig. 5A). Comparing only those hearts that returned to intrinsic rhythm, there was no significant differences between the initial EDP rise upon reperfusion in the 145 and 155 mM Na^+^ groups (Fig. 5B). Interestingly, in the 145 mM Na^+^ group, hearts that went on to remain asystolic or develop arrhythmias had a significantly higher EDP rise upon reperfusion. Although LVDP was decreased during reperfusion in both the 145 and 155 mM Na^+^ groups relative to baseline, LVDP during reperfusion was significantly higher in the 145 mM Na^+^ group relative to the 155 mM Na^+^ group (Fig. 5C). This is consistent with the pre-ischemic relationship between [Na^+^]_o_ and LVDP. Reperfusing hearts with elevated [Na^+^]_o_ did not significantly alter coronary flow rate (data not shown).

## Discussion

Previous studies from our laboratory suggest that elevating [Na^+^]_o_ in ex vivo hearts increases electrical stability during ischemia^2,3,20^. Historically, our studies were performed in contraction arrested hearts to reduce motion artifact in order to facilitate optical mapping; therefore, we have not previously studied the mechanical consequences of modifying perfusate ionic composition. This study demonstrates that elevating perfusate Na^+^ depresses cardiac mechanical function during baseline conditions and ischemia–reperfusion. Specifically, elevating [Na^+^]_o_ reduces LVDP in an NCX-dependent manner at baseline, accelerates the onset of ischemic contracture, and reduces LVDP during reperfusion.

### Baseline

The first evidence of an antagonistic relationship between Na^+^ and Ca^2+^ was provided over a half century ago in 1948^4–6^. Since the initial publications demonstrating Na^+^-Ca^2+^ exchange, the role of transmembrane Na^+^ currents on cardiac inotropy has been well-described^21,22^. More specifically, studies revealed that large changes in [Na^+^]_o_ significantly altered Ca^2+^ flux across the sarcolemma via NCX^8,9,23^. In these studies, reducing or completely eliminating [Na^+^]_o_ markedly increased cellular Ca^2+^ influx through reverse-mode NCX and increased inotropy. Conversely, elevating [Na^+^]_o_ reduces inotropy^12^. Importantly, it should be noted that these studies were all performed in isolated cells or relied on changing [Na^+^]_o_ to levels far beyond physiological concentrations. In our study, we demonstrated that a modest elevation in [Na^+^]_o_ to 155 mM is negatively inotropic, qualitatively consistent with previous reports using larger changes in [Na^+^]_o_^12^. Furthermore, we found that LVDP depression in the presence of elevated [Na^+^]_o_ was mitigated when NCX was inhibited prior to [Na^+^]_o_ elevation (Fig. 3). Together these data suggest elevating [Na^+^]_o_ at baseline results in a reduction of [Ca^2+^]_o_, via increased NCX Ca^2+^-efflux, and decreased contractility.

### Ischemia

To our knowledge, this is the first study to demonstrate that the time course of ischemic contracture can be modulated by perfusate ionic composition (Fig. 4). Specifically, elevating [Na^+^]_o_ in the pre-ischemia perfusate accelerates the onset of ischemic contracture and NCX inhibition further accelerates this process. Previous studies have demonstrated that ischemia induced increases in diastolic tone are correlated with intracellular ATP (ATP_i_) depletion and intracellular proton accumulation (H^+^_i_, i.e. acidosis), presumably by a mechanism of [Na^+^]_i_ mediated mitochondrial dysfunction^18,19,24–27^. Studies also provided evidence that slowing ATP depletion, by pre-treatment with dimethyl-α-ketoglutarate, delays the onset of contracture development^28^. These studies suggest preservation of whole-cell metabolic function delays the time to peak ischemic contracture.

Both ATP_i_ depletion and H^+^_i_ accumulation are hallmarks of mitochondria dysfunction within ischemic myocardium^29,30^. In fact, increasing [Na^+^]_o_ in isolated myocytes impairs the ability of mitochondria to adapt to stressors such as ischemia^18,27^. Given that ischemic contracture is dependent on ATP-depletion and increasing [Na^+^]_o_ worsens mitochondrial ischemic injury, it is not surprising that the onset of ischemic contracture occurred earlier in the elevated [Na^+^]_o_ perfusion group. Additionally, inhibiting NCX, which increases [Na^+^]_i_ by eliminating a major Na^+^-extrusion pathway during ischemia, further accelerated ischemic contracture in a manner consistent with the hypothesis that NCX function is an important determinant of ischemic contracture.

### Reperfusion

Numerous studies have demonstrated that ventricular arrhythmias and myofibrillar hypercontracture observed upon reperfusion are due, at least in part, to intracellular Ca^2+^ overload and increased CaMKII activity^10,11^. Interestingly, we found that hearts which returned to intrinsic rhythm during reperfusion presented with a significantly lessened degree of hypercontracture, as measured by reperfusion EDP (Fig. 5B). The decreased reperfusion contracture amplitude observed in intrinsic activity suggests that elevating [Na^+^]_o_ may mitigate ischemia and reperfusion injury, presumably by reducing the degree of intracellular Ca^2+^-overload^31–35^.

With respect to the return of mechanical function, for both 145 and 155 mM Na^+^, the absolute LVDP during reperfusion was lower than during baseline, and paralleled the differences observed at baseline. Several factors could have contributed to this relative decrease in LVDP. It is known that ATP availability is markedly reduced during prolonged ischemic episodes, and previous work by Maack et al. suggests elevating [Na^+^]_o_ hinders mitochondrial metabolic flexibility, resulting in impaired contractility^18,36^. Furthermore, during reperfusion, and similar to baseline conditions, elevated [Na^+^]_o_ can lead to increased NCX Ca^2+^-efflux and reduced inotropy via intracellular Ca^2+^ depletion. One additional explanation for decreased LVDP during reperfusion is that elevated [Na^+^]_o_ modulates vascular resistance during ischemia–reperfusion. This is important because previous work demonstrated LVDP is proportional to vascular pressure^37,38^. While an intriguing trend was observed, flow rates were not significantly decreased in hearts reperfused with elevated [Na +]_o_ post ischemia (p = 0.11) in this study. Further studies will be required to elucidate the effects of modulating vascular resistance by perfusate ion composition in a working heart during baseline conditions and ischemia–reperfusion.

With statistically significant differences in contractile function observed during baseline perfusion with our 145 and 155 mM Na^+^ perfusates (Table 2 and Fig. 2), it is possible that the reperfusion results are a manifestation of changes in [Na^+^]_i_ and/or [Ca^2+^]_i_ that occur during baseline. Future studies will need to assess whether the changes observed during reperfusion occur independent of the pre-ischemic perfusate. Regardless of the mechanism, the finding that upon ischemia–reperfusion LVDP is lowest in hearts perfused with 155 mM Na^+^ may warrant further investigation given the prevalent use of Normal Saline IV solutions, which contain 155 mM NaCl, in clinical practice.

### Limitations

Recently published ex vivo Langendorff perfusion guidelines recommend excision and cannulation be < 3 min, and many of our experiments were performed before the publication of these guidelines and instead used cannulation times of < 4 min as an inclusion criteria^39^. Furthermore, we measured left-ventricular pressure using latex balloons. Since latex has a known compliance, our measurements could underestimate left-ventricular pressure^40^.

The values of LVDP reported herein are lower than previously published studies. This is a consequence of our laboratory’s perfusate historically containing 1.25 mM Ca^2+^ in order to reduce motion for optical mapping in Langendorff perfused hearts^1,2^. Research groups that study LVDP and cardiac mechanics historically perfuse preparations with 1.8 mM Ca^2+^. Additionally, our perfusion pressure, 60 cm H_2_O (~ 45 mmHg), was lower than the traditionally used 60–80 mmHg.

Rat cardiomyocytes have documented differences in Ca^2+^ handling properties compared to humans and larger mammalian species. For example, rat NCX current is lower than I_NCX_ in larger mammalian species, which was elegantly demonstrated by Sham et al.^41^. Despite these known differences in Ca^2+^ handling, the general finding, that elevating [Na^+^]_o_ depresses cardiac contraction via an NCX mechanism, is likely applicable to other mammalian species.

## Conclusions

While elevating [Na^+^]_o_ may reduce arrhythmias during ischemia–reperfusion^2,3^, this study suggests the same intervention depresses LVDP and reinforces the tight and often opposing associations between electrophysiology and mechanical function with regards to outcome. More specifically, therapies that reduce arrhythmic events may be advantageous when depressed mechanical function is not pathologic. Alternatively, the study suggests that rescuing mechanical dysfunction during ischemia–reperfusion at the expense of enhanced arrhythmic risk may warrant further investigation if a shockable rhythm is preferable to a non-shockable rhythm without pump function.

Finally, and most broadly speaking, this work reveals that relatively small changes in ionic composition, particularly in isolated organ experiments, may produce substantially different response magnitudes, and these changes could explain why some therapies have significant and dramatic effects in a laboratory where extracellular ions are experimental regulated, but fail to produce the same dramatic response in humans when extracellular ionic composition is tightly controlled and dynamically modulated.

## Methods

All protocols were approved by the Institutional Animal Care and Use Committee of Virginia Polytechnic Institute and State University and were in accordance with the National Institutes of Health *Guide for the Care and Use of Laboratory Animals*.

### Langendorff heart preparation

Male Sprague–Dawley rats (8–12 weeks; 225–300 g) were anesthetized with ketamine/xylazine (90 mg/kg ketamine, 10 mg/kg xylazine, i.p.) without heparinization. Upon the absence of animal eye-blink and pedal withdraw response, hearts were excised and quickly cannulated (< 4 min) on a constant pressure (60 cm H_2_O) Langendorff apparatus. Hearts were perfused with a bicarbonate buffered perfusate containing either 145 or 155 mM Na^+^ (full composition listed in Table 1) at approximately 37 °C. Hearts were submerged in a 3D printed PLA bath for superfusion as previously described^42^. Hearts remained submerged throughout global ischemia to maintain organ temperature. Coronary flow rate and a volume conducted electrocardiogram were recorded continuously throughout the experiment. To measure left-ventricular function, a size 4 latex isovolumetric pressure balloon (Harvard Apparatus catalog #73-0303) was inserted into the left ventricle through a small slit created in the left atria. The balloon was inflated to a baseline diastolic pressure between 0 and 10 mmHg, as previously described^43^. End-diastolic pressure (EDP), end-systolic pressure (ESP), and left-ventricular developed pressure (LVDP) were recorded continuously throughout the experiment using an ADInstruments Powerlab 4/26 data acquisition system and LabChart recording software.

**Table 1:** Detailed composition of perfusates used in this manuscript.

	Concentration in mM							pH
	Total Na^+^	NaCl	NaHCO_3_	KCl	Dextrose	MgCl_2_∙6H_2_O	CaCl_2_∙2H_2_O	
145 Na^+^	145.0	122.0	23.0	4.6	5.5	0.7	1.25	7.40 ± 0.02
155 Na^+^	155.0	132.0	23.0	4.6	5.5	0.7	1.25	7.40 ± 0.02

All perfusion sequences used in this experiment are presented in Fig. 1. Following cannulation and balloon positioning, hearts were left unperturbed on the Langendorff-apparatus for a 30-min stabilization and baseline measurement period, prior to 30 min of global no-flow ischemia and 30 min of reperfusion. In hearts subjected to sequential perfusion (data presented in Fig. 2), all hearts were cannulated first using the 145 mM Na^+^ perfusate and perfused for 15-min. At 15-min, the perfusate was switched to the 155 mM Na^+^ perfusate for the duration of the experiment. Baseline data reported in Fig. 2 were collected in the last 5-min of the baseline period (t = 25–30 min). Data reported in Table 2 were collected in the last 5-min of each group (t = 10–15 min and t = 25–30 min). LVDP, flow rate, and heart rate for each heart was reported as the average LVDP, flow rate, and heart rate during the final 5 min of the baseline period. In Fig. 5, asystolic hearts were defined as hearts which did not have any electrical activity detectable on the electrocardiogram during reperfusion. VT/VF hearts were defined as those hearts with sustained ventricular arrhythmias. In all three of these hearts, once initiated, VT/VF was sustained for the remainder of the experiment. Intrinsic rhythm was defined as any heart with a spontaneous return of intrinsic rhythm within the first 3-min of reperfusion and persisted arrhythmia-free (no more than 3-min of total sustained ventricular arrhythmias) throughout the 30-min reperfusion period. Reperfusion contracture was quantified as the peak EDP occurring immediately upon reperfusion as indicated in Fig. 1 and always occurred before the restoration of electrical activity, regardless of the eventual electrical outcomes.

**Table 2:** Summary of end-diastolic pressure (EDP), left-ventricular developed pressure (LVDP), flow rate (FR), and heart rate (HR) during the baseline perfusion period.

	n	EDP (mmHg)	LVDP (mmHg)	FR (mL/min)	HR (bpm)
145 Na^+^	16	6.3 ± 5.2	50.6 ± 16.8	11.2 ± 5.9	273.6 ± 57.4
155 Na^+^	13	6.4 ± 7.0	28.5 ± 10.6*	12.2 ± 8.1	277.4 ± 61.3

All measurements were obtained 25-min post-cannulation.

*Indicates p < 0.05 as determined by unpaired student’s t-test.

Hearts were excluded if cannulation took > 4 min, a sustained ventricular arrhythmia developed during the stabilization period, or if the isovolumetric balloon became displaced from the left ventricle at any point during the experiment.

### NCX inhibition with SEA0400

In a subset of experiments, NCX was inhibited with SEA0400 (1 µM). SEA0400 is a selective inhibitor of NCX demonstrated to inhibit both forward-mode and reverse-mode NCX activity; at a 1 µM concentration, SEA0400 effectively inhibits NCX activity without affecting other sarcolemma channels, pumps, or exchangers^15,16^. In these experiments, hearts were cannulated and the pressure balloon inserted using the 145 mM Na^+^ perfusate and left unperturbed for a 15-min stabilization period. Hearts were then perfused with the 145 Na^+^ perfusate + 1 µM SEA0400 for 15-min. Next, hearts were perfused with 155 Na^+^ perfusate + 1 µM SEA0400 for 15-min. Average LVDP during the last five minutes was quantified for each group. After baseline LVDP values were recorded, hearts were subjected to 30-min of no-flow global ischemia.

### Statistical analysis

Statistical analyses were performed using GraphPad Prism 7. For all data, p < 0.05 was considered statistically significant. Data from a total of 36 hearts are reported in this study. Specific n-values for each experimental group are included in the figure legends. All summary data are presented as mean ± standard error unless otherwise noted. Data in Figs. 2 and 3 were analyzed using a paired two-tailed Student’s t-test. Data in Table 2 were analyzed using an unpaired two-tailed Student’s t-test. Data in Fig. 4 were analyzed using a one-way ANOVA with Dunnett’s correction for multiple comparisons with the155 Na^+^ group serving as the comparison group. In Fig. 5A, a Chi-square analysis was used for reperfusion arrhythmia comparisons. In Fig. 5B,C, data were analyzed using an unpaired two-tailed Student’s t-test.

A subset of experiments were performed in a blinded fashion by a second experimentalist to confirm results. The blinded experimentalist performed a total of 12 experiments. (n = 3 and n = 3 for 145 Na^+^ and 155 Na^+^ respectively, data combined in Table 2. n = 3 and n = 3 for 145 Na^+^ and 155 Na^+^ respectively, data combined in Figs. 4 and 5).
